# Course of disease and risk factors for hospitalization in outpatients with a SARS-CoV-2 infection

**DOI:** 10.1038/s41598-022-11103-0

**Published:** 2022-05-04

**Authors:** Eik Schäfer, Christian Scheer, Karen Saljé, Anja Fritz, Thomas Kohlmann, Nils-Olaf Hübner, Matthias Napp, Lizon Fiedler-Lacombe, Dana Stahl, Bernhard Rauch, Matthias Nauck, Uwe Völker, Stephan Felix, Guglielmo Lucchese, Agnes Flöel, Stefan Engeli, Wolfgang Hoffmann, Klaus Hahnenkamp, Mladen V. Tzvetkov

**Affiliations:** 1 Department of Clinical Pharmacology, Institute of Pharmacology, Center of Drug Absorption and Transport (C_DAT), University Medicine Greifswald, Greifswald, Germany; 2 Department of Anesthesiology, University Medicine Greifswald, Greifswald, Germany; 3 Department of General Pharmacology, Institute of Pharmacology, Center of Drug Absorption and Transport (C_DAT), University Medicine Greifswald, 17489 Greifswald, Germany; 4 Institute for Community Medicine, Section Epidemiology of Health Care and Community Health, University Medicine Greifswald, Greifswald, Germany; 5 Central Unit for Infection Prevention and Control, University Medicine Greifswald, Greifswald, Germany; 6 Institute of Hygiene and Environmental Medicine, University of Greifswald, Greifswald, Germany; 7 Departments of Emergency and Acute Medicine, University Medicine Greifswald, Greifswald, Germany; 8 Independent Trusted Third Party, University Medicine Greifswald, Greifswald, Germany; 9 Institute of Clinical Chemistry and Laboratory Medicine, University Medicine Greifswald, Greifswald, Germany; 10 DZHK (German Center for Cardiovascular Research), Partner Site Greifswald, Greifswald, Germany; 11 Department of Functional Genomics, University Medicine Greifswald, Greifswald, Germany; 12 Department of Internal Medicine B, Cardiology, Pneumology, Infectious Diseases, Intensive Care Medicine, University Medicine Greifswald, Greifswald, Germany; 13 Department of Neurology, University Medicine Greifswald, Greifswald, Germany

**Keywords:** Diseases, Health care, Medical research, Risk factors

## Abstract

We analyzed symptoms and comorbidities as predictors of hospitalization in 710 outpatients in North-East Germany with PCR-confirmed SARS-CoV-2 infection. During the first 3 days of infection, commonly reported symptoms were fatigue (71.8%), arthralgia/myalgia (56.8%), headache (55.1%), and dry cough (51.8%). Loss of smell (anosmia), loss of taste (ageusia), dyspnea, and productive cough were reported with an onset of 4 days. Anosmia or ageusia were reported by only 18% of the participants at day one, but up to 49% between days 7 and 9. Not all participants who reported ageusia also reported anosmia. Individuals suffering from ageusia without anosmia were at highest risk of hospitalization (OR 6.8, 95% CI 2.5–18.1). They also experienced more commonly dyspnea and nausea (OR of 3.0, 2.9, respectively) suggesting pathophysiological connections between these symptoms. Other symptoms significantly associated with increased risk of hospitalization were dyspnea, vomiting, and fever. Among basic parameters and comorbidities, age > 60 years, COPD, prior stroke, diabetes, kidney and cardiac diseases were also associated with increased risk of hospitalization. In conclusion, due to the delayed onset, ageusia and anosmia may be of limited use in differential diagnosis of SARS-CoV-2. However, differentiation between ageusia and anosmia may be useful for evaluating risk for hospitalization.

## Introduction

Thus far, SARS-CoV-2 infections have caused more than 6.2 million deaths worldwide^1^ and despite the development of effective vaccines, these infections still heavily influence daily life, economic activity and society worldwide.

Early diagnosis of a SARS-CoV-2 infection is pivotal to control the spread of the virus, pointing to the importance of the identification of specific symptoms with early manifestations. Fever, fatigue, cough, loss of smell and loss of taste are known symptoms of a SARS-CoV-2 infection^2,3^. However, most of the symptoms are nonspecific and may occur with different common viral infections. Furthermore, we need systematically collected data about the frequency and the time of onset of the symptoms of SARS-CoV-2 infection.

The severity of SARS-CoV-2 infection varies broadly among infected individuals and is difficult to predict in advance. Infected individuals may be asymptomatic, develop mild to moderate symptoms that do not require hospitalization, or need specialized medical care. Older age, male sex, and chronic kidney or cardiovascular disease are more commonly associated with a severe course of the disease and hospitalization^4,5^. Symptoms like fever, cough and dyspnea were reported to be more common in patients who needed hospitalization^6^.

Less than 10% of infected individuals are admitted to a hospital^7^. Out of them, about a quarter died in the hospital or during the immediate post-discharge period^8,9^. The course of the infection in hospitalized patients is well described. The first detailed report was published by Zhu et al.^10^ and descriptions of severe disease courses have rapidly accumulated ever since.

Scores like 4C or CIRC (COVID-19 Inpatient Risk Calculator) have been developed to predict severity or mortality in COVID-19 patients after admission to a hospital^11–14^. In their prediction, they include demographic factors, comorbidities, and even symptoms, but they are not directly transferable to outpatient setting.

However, the majority of the infected individuals (above 90%) do not require hospitalization. Prospective data collections in these patients are still rare, although they might help to establish an early diagnosis. Furthermore, these data should help to identify factors predicting hospitalization later in the course of the disease.

Here we report an observational cohort study on the course of SARS-CoV-2 infections in more than 700 outpatients in Germany. This study characterizes the course of symptoms during 25 days after infection aiming to identify specific symptoms and time patterns. We also analyzed symptoms and comorbidities as predictors for hospitalization.

## Methods

### Study design and participants

We performed a prospective observational cohort study of SARS-CoV-2 positive outpatients. With the help of the state office of health in the district of Vorpommern-Greifswald, Germany, we invited adults (18 years of age or older) with a PCR-confirmed SARS-CoV-2 infection to participate in our study. Study participants were asked to provide informed consent and to fill out two consecutive questionnaires. The first questionnaire pertained to demographics and comorbidities. The second one was a symptom diary covering the symptoms within the 25 days after the start of the observational period. The start of the observational period was defined either as the day of occurrence of the first symptoms (n = 612; 87.4%), or as the day of the positive PCR test (n = 56; 7.7%). The two questionnaires were sent together with an information sheet and a sheet with a brief overview about the study. Prepaid, pre-addressed envelopes were provided to return the documents back to the study center. For further information about our study, participants were invited to contact us by phone or e-mail. The only exclusion criterion was absence of a valid informed consent.

Our primary objective was to identify factors that may negatively influence the course of SARS-CoV-2 infections and lead to hospitalization in outpatients. The secondary objective was to describe the course of symptoms within the acute phase of the infection in patients with mild or moderate SARS-CoV-2 infection. The study was conducted in accordance with the 2013 Declaration of Helsinki and was approved by the Ethics Committee of the University Medicine Greifswald (internal Ethics Committee No.: BB 059/20). This study is registered in the German Clinical Trials Register (DRKS00021672).

To increase the power of identifying risk factors for hospitalization, we additionally included 143 participants hospitalized and treated for COVID-19 at Greifswald University Hospital, the largest hospital in the district of Vorpommern-Greifswald (ViP study, DRKS ID: DRKS00023770). The participants were recruited after hospitalization. ViP is an observation study to collect blood samples and medical data, such as previous medical conditions and current medication. Furthermore, medical data during the hospital stay, such as therapy-related medication and mechanical ventilation was collected.

### Questionnaire

We developed a two-part questionnaire: one to collect possible risk factors present already before the infection and the second one in the form of a diary to self-document SARS-CoV-2 related symptoms over the first 25 days during the acute phase of the infection.

The first part of the questionnaire contained sociodemographic variables, such as gender, age, weight and pre-existing co-morbidities such as other chronic bacterial or viral infections, current medication, vaccination status, nutritional supplements and daily life activity. If a pre-existing disease was ticked “yes”, the participants could provide additional information. For this purpose, a detailed list of diseases was provided both in layman and medical terms, including heart diseases (coronary heart disease, heart failure, myocardial infarction), high blood pressure (systolic blood pressure before infection in different ranges), peripheral artery disease, diabetes mellitus (if known, HbA1c value in %), pulmonary diseases (asthma, COPD, obstructive sleep apnea), allergies (house dust allergy, allergic rhinitis), neurological diseases (stroke, multiple sclerosis, Parkinson’s disease, dementia), liver diseases (fatty liver, liver cirrhosis), renal diseases (dialysis, chronic kidney disease), joint diseases (rheumatoid arthritis, gout) and cancer of the lung, blood, breast or colon. It was possible to add other diseases within each organ system.

The second part of the questionnaire contained a table of 16 symptoms to be completed by the participant daily over 25 days after the onset of the initial symptoms. The symptoms were fever, dyspnea, dry cough, productive cough, headache, myalgia/arthralgia, fatigue, anosmia, ageusia, sore throat, conjunctivitis, skin manifestation, abdominal pain, diarrhea, nausea, and vomiting. When no symptoms had occurred before receiving our questionnaire, the day of the positive test was nominated to start with the table. In addition, we asked about the use of medication for the symptoms of COVID-19 and whether hospitalization was required. The questionnaire was provided in German language.

### Data management and statistical analyses

Informed consent was checked by the Independent Trusted Third Party of the University Medicine Greifswald. The identifying personal data was removed from the questionnaires, which were pseudonymized and then forwarded to the study center where they were entered into the database. Manual double data entry was used, followed by a quality check of the completed database. The participants were re-contacted via telephone in cases where data about hospitalization (primary endpoint) was missing, or if the participants returned the symptom diary before day 25, to solicit information about hospitalization connected to the infection. Regarding the symptom diary, missing data was imputed as “symptom not present” except if (i) symptoms reporting was discontinued for all symptoms by the participant before day 25, or (ii) the participant did not start reporting symptoms on the first day of the observation period. In these cases, we did not perform imputation and regarded the data as missing.

Statistical analyses were performed using IBM SPSS Statistics version 26. Medians and quartiles are used as descriptive statistics for continuous variables. Differences between groups of continuous variables were assessed using Mann–Whitney-U tests. Categorical variables are given in absolute numbers and percentages and differences in the distribution were assessed using Pearson’s χ^2^ test. All the tests used where two-tailed. Binary logistic regression was used for multivariable analyses of effects on hospitalization.

## Results

### Characteristics of the study population

Between May 2020 and February 2021, we invited 3840 individuals who tested positive for SARS-CoV-2 to participate in the study. These represent 69.7% of all infections from the district of Vorpommern-Greifswald in the northeast of Germany (total population of 235,773) during the second infection wave (Supplementary Fig. 1). The remaining 30.3% were not invited because they were younger than 18 years, had missing address data, or their positive test result was older than 1 week. Nine-hundred-eighty-three individuals (25.6% of all invited) agreed to participate in the study, and 710 returned both questionnaires and were included in the analyses. Population characteristics and comorbidities are described in Table 1.

**Table 1:** Basic characteristics and risk factors at baseline of the study population.

Parameter	All (n = 710)	Male (n = 285)	Female (n = 425)	p-value*
Age in years	53 (40–62)	55 (39–63)	52 (40–60)	0.067
BMI in kg/m^2^	26.6 (23.7–29.9)	27.5 (24.7–30.5)	25.9 (23.0–29.6)	3.4 × 10^–4^
Grade of disability in %	50 (30–60)	50 (40–70)	50 (30–60)	0.070
Current smoker	116 (16.3%)	45 (15.8%)	71 (16.7%)	0.746
Former smoker	211 (29.7%)	120 (42.1%)	91 (21.4%)	3.3 × 10^–9^
**Participants with at least one comorbidity (%)**	489 (68.9%)	201 (70.5%)	288 (67.8%)	0.436
Hypertension	264 (37.2%)	117 (41.1%)	147 (34.6%)	0.081
Allergy	144 (20.3%)	49 (17.2%)	95 (22.4%)	0.094
Joint diseases	133 (18.7%)	55 (19.3%)	78 (18.4%)	0.752
Cardiovascular diseases	80 (11.3%)	47 (16.5%)	33 (7.8%)	3.1 × 10^–4^
Pulmonary diseases	64 (9.0%)	33 (11.6%)	31 (7.3%)	0.051
Diabetes mellitus	61 (8.6%)	35 (12.3%)	26 (6.1%)	0.004
Neoplasia	56 (7.9%)	28 (9.8%)	28 (6.6%)	0.117
Neurological diseases	50 (7.0%)	21 (7.4%)	29 (6.8%)	0.781
Liver diseases	49 (6.9%)	16 (5.6%)	33 (7.8%)	0.268
Kidney diseases	33 (4.6%)	15 (5.3%)	18 (4.2%)	0.526
Peripheral artery disease	14 (2.0%)	8 (2.8%)	6 (1.4%)	0.190
Participants with disability	138 (19.4%)	62 (21.8%)	76 (17.9%)	0.201

Shown are median (first and third quartile) for age, BMI and grade of disability and absolute number (prevalence) for the remaining variables.

*For differences between male and female participants (calculated using Pearson χ^2^ test except for age, BMI and grade of disability where Mann–Whitney U test was used).

### Frequency, duration and time course of the infection symptoms

We asked patients to monitor 16 infection symptoms (Table 2). We received information about the symptoms from 700 out of 710 participants.

**Table 2:** Reported frequency, time of onset and persistence of symptoms.

Symptom	Number of individuals (% of all who answered) with the symptom			Median day of onset (range)*	Mean duration (days)
	At least 1 day	5 or more days	10 or more days		
Fatigue	557 (79.6%)	471 (67.3%)	314 (44.9%)	2 (1–15)	12.1
Headache	487 (69.6%)	314 (44.9%)	151 (21.6%)	3 (1–20)	7.5
Dry cough	456 (65.1%)	360 (51.4%)	218 (31.1%)	3 (1–16)	10.6
Myalgia/arthralgia	443 (63.3%)	303 (43.3%)	128 (18.3%)	2 (1–20)	7.7
Loss of smell	375 (53.6%)	317 (45.3%)	195 (27.9%)	4 (1–13)	11.6
Loss of taste	351 (50.1%)	290 (41.4%)	161 (23.0%)	4 (1–17)	10.6
Sore throat	327 (46.7%)	165 (23.6%)	68 (9.7%)	3 (1–24)	6.3
Productive cough	251 (35.9%)	161 (23.0%)	102 (14.6%)	5 (1–24)	8.9
Diarrhea	245 (35.0%)	76 (10.9%)	19 (2.7%)	4 (1–23)	3.8
Dyspnea	224 (32.0%)	168 (24.0%)	115 (16.4%)	4 (1–17)	11.2
Nausea	206 (29.4%)	81 (11.6%)	24 (3.4%)	4 (1–21)	4.6
Abdominal pain	180 (25.7%)	58 (8.3%)	23 (3.3%)	4 (1–23)	4.6
Fever ≥ 38 °C	180 (25.7%)	39 (5.6%)	5 (0.7%)	2 (1–21)	3.2
Skin manifestation	72 (10.3%)	47 (6.7%)	23 (3.3%)	7 (1–20)	8.0
Conjunctivitis	54 (7.7%)	25 (3.6%)	7 (1.0%)	7.5 (1–23)	5.3
Vomiting	43 (6.1%)	3 (0.4%)	2 (0.3%)	6 (1–22)	2.2

Symptoms are sorted according to their frequency starting with the most frequent one.

*In relation to the onset of the first symptom, or in the case of an asymptomatic begin of the infection of the day of the positive PCR-test.

**Figure 1:**
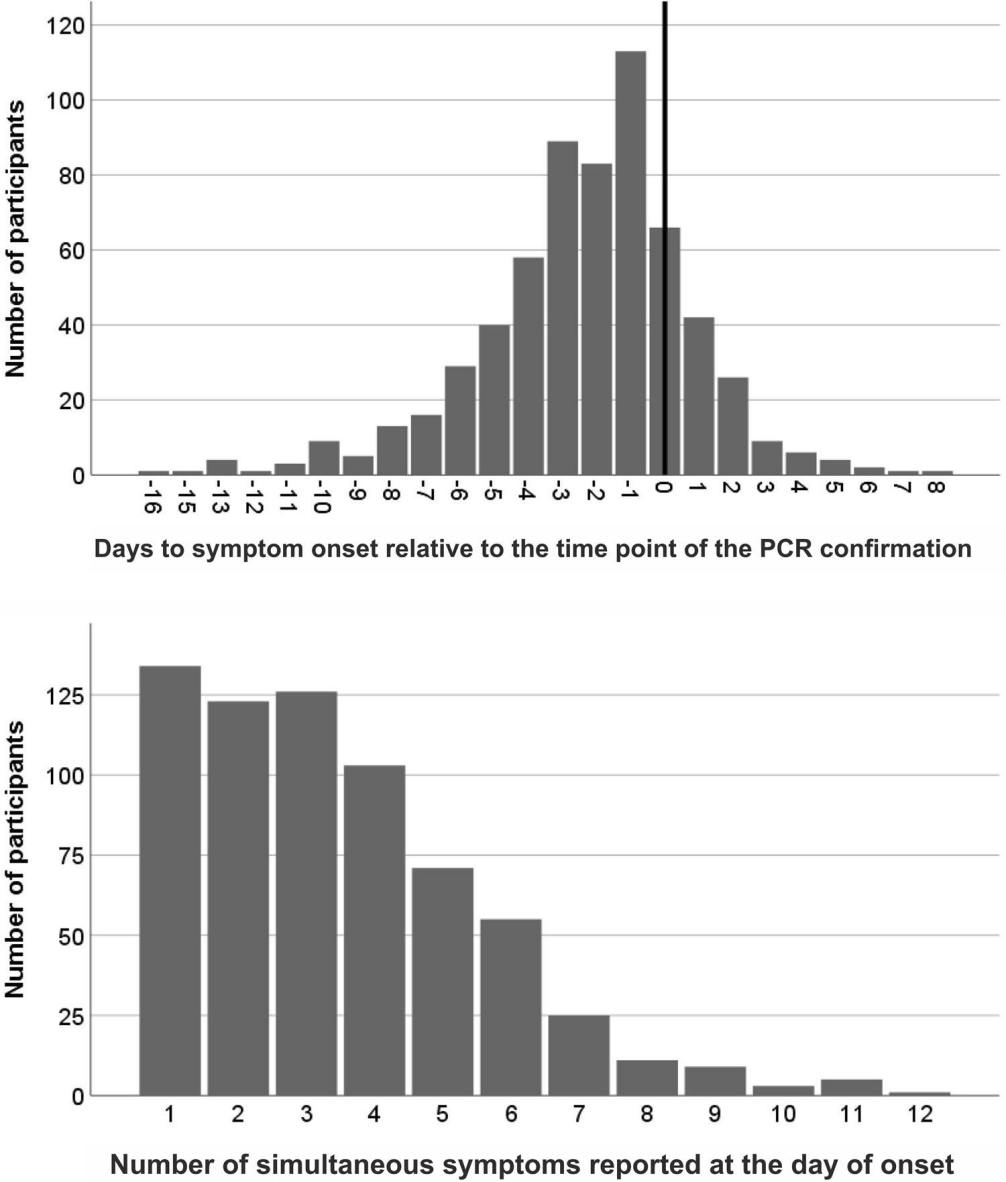
Time of onset of symptoms and number of symptoms on the day of onset. *Upper panel* shows the day of onset of symptoms relative to the day of infection confirmation via PCR (day 0 is set as the day of taking the sample for the PCR test) of all 622 participants that self-reported the precise time of symptom onset. 91 individuals did not report any symptoms on the day of the PCR test. *Lower panel* shows the number of simultaneous symptoms at the day of onset of the first symptom.

Thirty-four individuals (4.9%) self-reported to have been completely asymptomatic during the entire observation period. Of the remaining participants, 622 reported the exact time point of the symptom onset related to the infection confirmation via PCR (Fig. 1: upper panel). The onset occurred on average 2.3 days before the confirmation of the infection by PCR test. Among the individuals with symptoms before the positive test result, 18.2% experienced the first symptom only one day before the test.

At symptom onset, participants reported a median of three simultaneous symptoms with a range from 1 (134 participants) to 12 symptoms (1 participant, Fig. 1, lower panel). Importantly, the reported initial symptoms were nonspecific, e.g. fatigue (60.3%), headache (48.6%), myalgia/arthralgia (46.1%) or sore throat (29.3%, Supplementary Table 1). More SARS-CoV-2-specific symptoms, e.g. loss of smell or loss of taste, were reported by only 15.4% and 13.7%, respectively, on the day of symptom onset.

**Figure 2:**
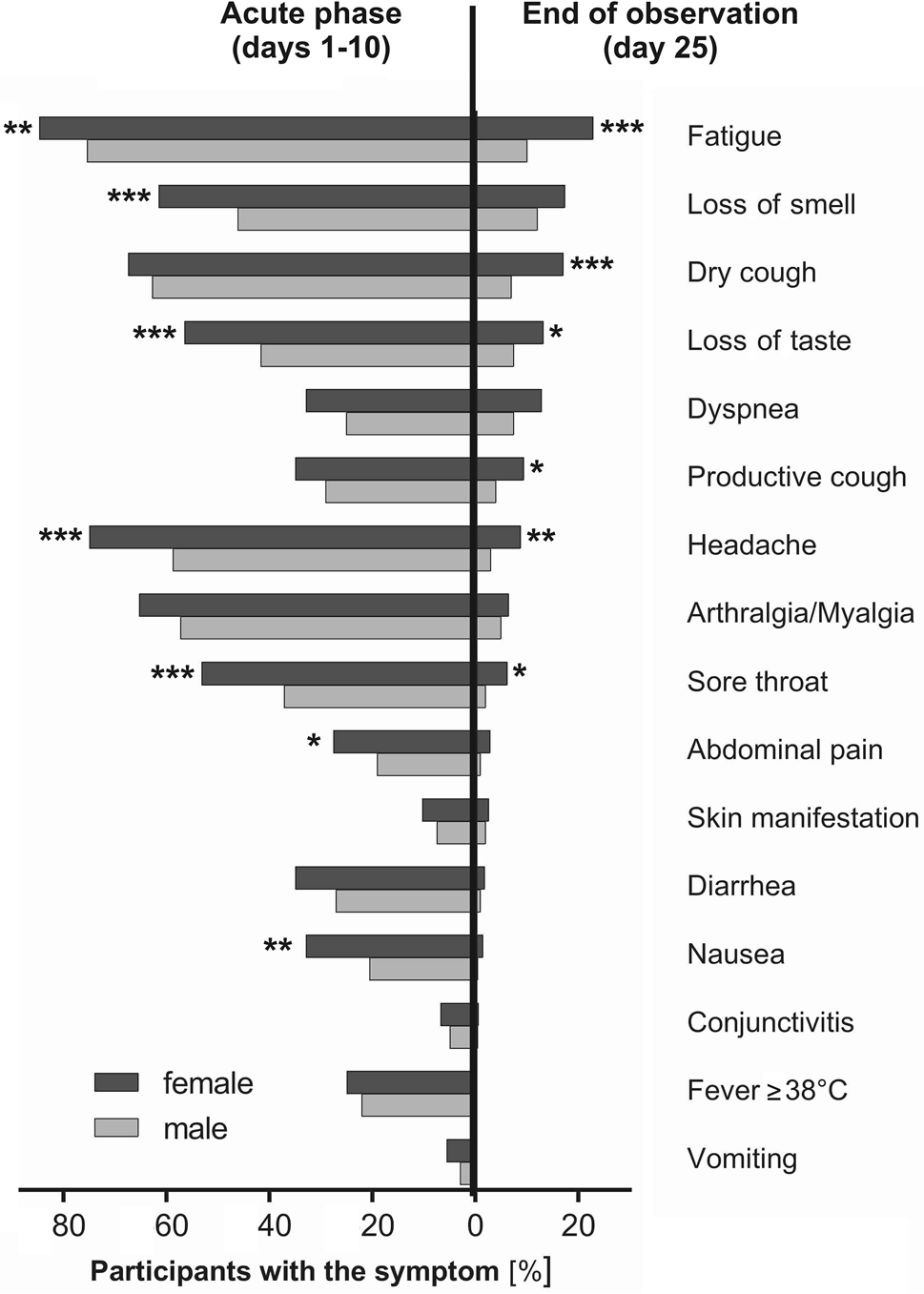
Comparative representation of the frequency of symptoms in the acute phase of the infection (days 1 to 10) and at the end of the observation period (day 25). Symptoms are sorted according to their persistence at day 25. The analyses are based on 539 participants who reported symptoms over the entire 25-day period. Shown are the frequencies of symptoms in female (n = 420, dark gray) and male (n = 280, white gray) participants. *p < 0.05, **p < 0.01, ***p < 0.001 for sex dependent differences calculated Pearson χ^2^ test.

Fatigue was the most persistent symptom (mean duration of 12.1 days), followed by loss of smell, dyspnea, loss of taste and dry cough (each lasting more than 10 days on average, Table 2). In contrast, the symptoms with the shortest persistence were vomiting, fever, diarrhea, nausea and abdominal pain (each with mean duration below 5 days, Table 2). Almost half of the participants (n = 239; 44.3%) reported at least one infection-related symptom on the last day of observation (day 25). Fatigue was the most frequently reported symptom on day 25 (n = 98, 18.2%, Fig. 2), followed by loss of smell (n = 83, 15.4%) and dry cough (n = 72, 13.4%; Fig. 2).

The frequency and duration of symptoms were sex-dependent. Women reported between 12 and 60% more commonly fatigue, loss of smell, loss of taste, headache, sore throat, abdominal pain, and nausea in the first 10 days of the infection and 23% of the women were still reporting fatigue at day 25 of the infection (Fig. 2, Supplementary Table 2).

The frequency and duration of some symptoms were also age-dependent. Younger participants (18–39 years) had shorter duration of fatigue, fever, arthralgia/myalgia, cough in any form, and skin lesions (Supplementary Table 4). On the other hand, fewer participants age 60 or above reported loss of smell and taste, headache, or sore throat (Supplementary Table 5), but when these symptoms occurred, their duration was not age-dependent (Supplementary Table 4).

### Course of symptoms

**Figure 3:**
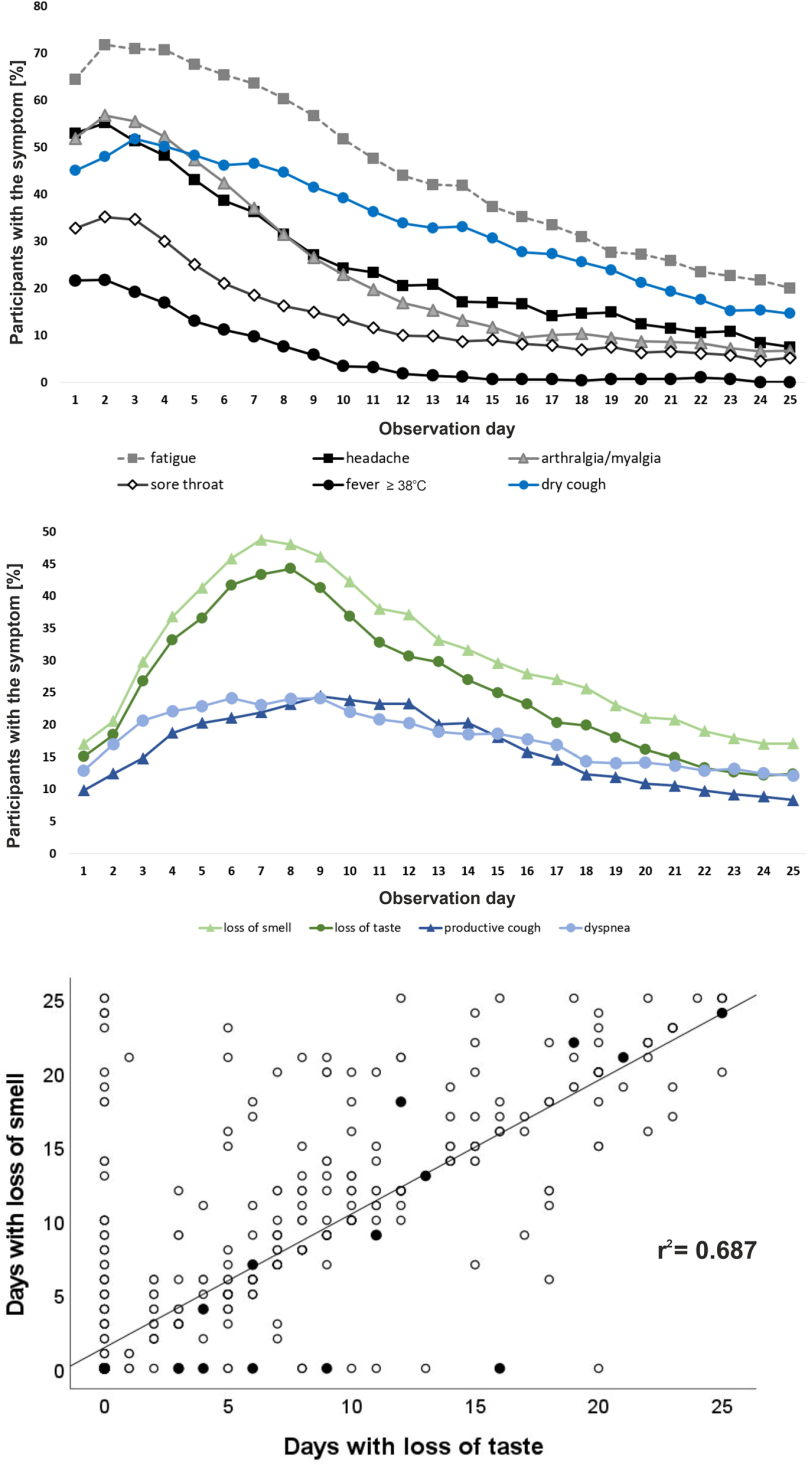
Course of SARS-CoV-2 symptoms within the 25 day observational period. Shown are 10 of the analyzed 16 symptoms stratified according to their specific pattern: strongest initially (*upper panel*) and increasing until days 7 to 9 (*middle panel*). General symptoms are shown in gray, neurological in green, and pulmonary in blue. Skin manifestations, conjunctivitis, abdominal pain, diarrhea, nausea and vomiting are not shown because fewer than 15% of participants reported these symptoms on any observation day. The figures represent frequencies based on the original (non-imputed) data. The results using the imputed data are quite similar with the exception of fever as body temperature was much less frequently documented by the participants. The *lower panel* shows the correlation between duration of loss of taste and smell. Each individual is represented as a single dot. Hospitalized participants are represented in filled and non-hospitalized in open circles.

We observed two distinct patterns in the time course of symptoms. The majority of the symptoms were reported within the initial three days of the infection and the percentage of individuals reporting them declined consistently with time (Fig. 3, upper panel). Within this group, the most rapidly improving symptom was fever, with fewer than 2% of the participants reporting fever after day 12.

The second pattern is characterized by steadily increasing frequencies among the participants in the first week, reaching the peak at days 7 to 9 (Fig. 3, middle panel). Interestingly, the symptoms regarded as characteristic for the disease like loss of smell and taste^15,16^ followed this alternative pattern in our study. At day one only 17.1% of the participants reported loss of smell or loss of taste, whereas 48.8% of the participants suffered from loss of smell on day 7 (Fig. 3, middle panel).

### Correlations between symptoms

The strongest correlation was observed between the duration of loss of smell and loss of taste (r = 0.83, p < 0.001, Supplementary Fig. 2). However, these two symptoms differed in duration. Loss of smell was reported for a longer duration (mean 11.6 days, median 10 days) compared to loss of taste (mean 10.6 days, median 9 days; p = 1.2 × 10^–8^ paired t-test). Importantly, 50 individuals reported loss of smell without loss of taste and 26 individuals reported loss of taste without loss of smell (Fig. 3, lower panel).

Strong correlations were also observed between the duration of general symptoms (fatigue and arthralgia/myalgia with r = 0.56 and fatigue and headache with r = 0.51, Supplementary Fig. 2).

Correlations were observed within the groups of specific symptoms (e.g. between nausea and abdominal pain and between diarrhea and vomiting), but also between the specific and unspecific symptoms, especially between the general and pulmonary symptoms like dyspnea and fatigue (r = 0.43, p < 0.001, Supplementary Fig. 2). Interestingly, days with fever only moderately correlated with any of the other symptoms documented (r < 0.3, Supplementary Fig. 2).

### Association of symptoms with hospitalization

Next, we analyzed whether symptoms in outpatients may be predictive for the risk of hospitalization. Data about symptoms and hospitalization due to the infection were available from 685 study participants, of which 36 participants (5.3%) were hospitalized. The median time between onset of the symptoms and hospitalization was 6 days (range 1 to 23 days).

**Figure 4:**
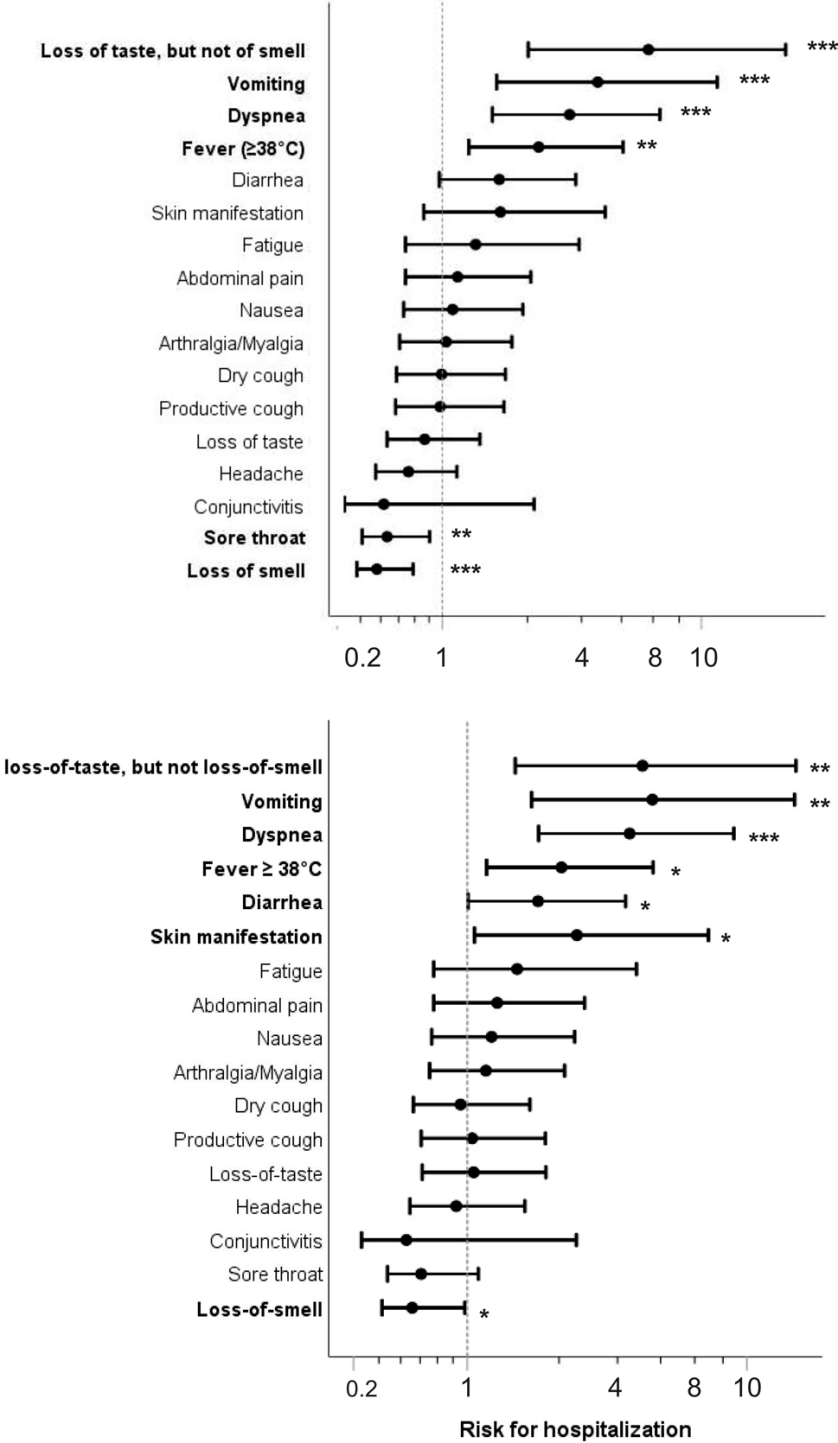
Association of symptoms with risk for hospitalization. Shown are the odds ratios and 95% confidence intervals without (upper panel) or after adjustment for age, sex and BMI (lower panel). Statistically significant associations are highlighted in bold and the levels of significance are reported (*p < 0.05, **p < 0.01, ***p < 0.001). The Pearson χ^2^ test was used to calculate pairwise two-sided significances. Adjustment for age, sex and BMI was performed using binary logistic regression with hospitalization as the dependent variable and including the three adjustment parameters and each of the symptoms separately as dependent variables.

Five symptoms were individually significantly associated with hospitalization (Fig. 4, upper panel). Vomiting, dyspnea and fever were associated with increased risk of hospitalization, while loss of smell and sore throat were associated with decreased risk (Fig. 4, upper panel).

**Figure 5:**
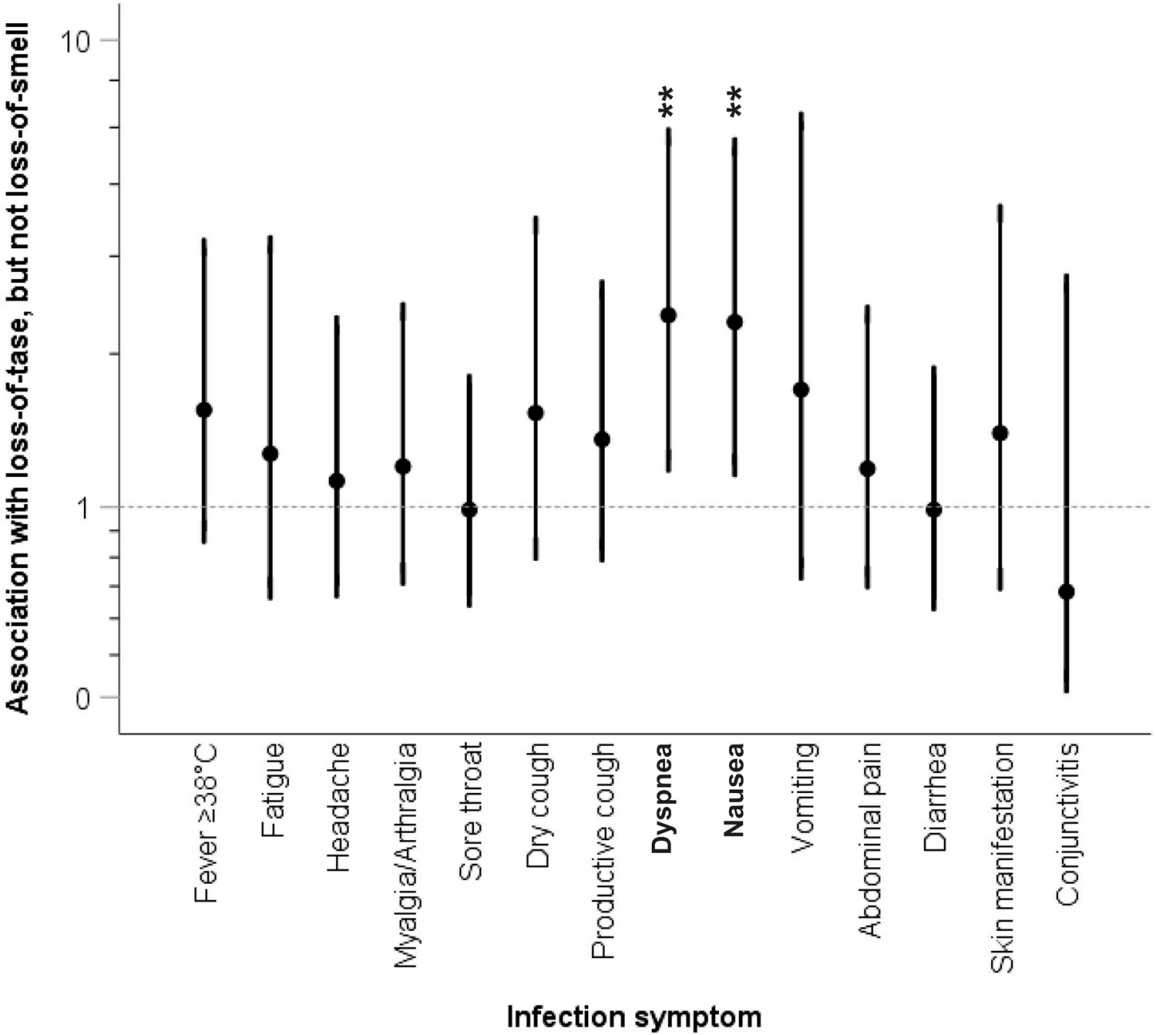
Association of loss of taste without loss of smell with other infection symptoms. Shown are the odds ratios and 95% confidence intervals for experiencing one of the other infection symptoms if the patient also suffered from loss of taste without loss of smell. Statistically significant associations are highlighted in bold and the levels of significance are reported (**p < 0.01). The Pearson χ^2^ test was used to calculate pairwise two-sided significances.

Importantly, individuals who reported loss of taste without loss of smell (n = 26) were at close to sevenfold higher risk of hospitalization (Fig. 4, upper panel). The association remained significant after adjustment for age, sex and BMI (Fig. 4, lower panel). Furthermore, individuals who reported loss of taste without loss of smell more commonly experienced dyspnea and nausea (Fig. 5).

### Association between risk factors at baseline with hospitalization

We also analyzed potential risk factors (demographic factors, comorbidities, and baseline medications) for associations with hospitalization. To enrich the participants in our study (36 participants hospitalized due to the infection), we analyzed 143 individuals hospitalized with COVID-19 in the University Hospital Greifswald (Supplementary Table 6), the major tertiary hospital in the region (ViP study, DRKS-ID: DRKS00023770). The resulting combined population included 838 persons infected with SARS-CoV-2, of which 179 (21.4%) participants were hospitalized due to the infection.

Age and BMI were highly significantly associated with risk of hospitalization (p < 10^–26^ and p = 0.004, respectively, Mann–Whitney-U Test; Supplementary Fig. 3).

In addition to age above 60 years, male sex and the comorbidities cardiovascular disease, hypertension, diabetes mellitus, pulmonary disease (both general pulmonary disease and COPD specifically), neurological disease (both general neurological disease and stroke specifically), and kidney disease were associated with increased risk of hospitalization (Table 3). Of these, age, neurological disease, COPD, cardiac disease, and diabetes were independent predictors of hospitalization (Table 3). Liver diseases, joint diseases, allergy and neoplasia were not significantly associated with the risk of hospitalization.

**Table 3:** Comorbidities as risk factors for hospitalization.

Risk factor	Individuals with this risk factor*	Univariate analyses		Multivariate analyses***	
		OR (95% CI)	p-value	OR (95% CI)	p-value
Age ≥ 60	329 (39.5%)	5.66 (3.93–8.15)	< 10^–20^	3.52 (2.02–6.20)	1.3 × 10^–5^
Male sex	365 (43.6%)	2.31 (1.65–3.24)	8.0 × 10^–7^		
Cardiovascular diseases	132 (15.9%)	4.70 (3.16–6.99)	6.5 × 10^–16^	1.84 (1.05–3.23)	0.03
Hypertension	345 (41.4%)	2.47 (1.75–3.46)	1.3 × 10^–7^		
Peripheral artery disease	17 (2.0%)	4.47 (1.70–11.8)	0.001		
Diabetes mellitus	94 (11.3%)	4.54 (2.90–7.10)	1.6 × 10^–12^	2.41 (1.32–4.39)	0.004
Pulmonary disease	97 (11.6%)	2.68 (1.71–4.20)	1.0 × 10^–5^		
COPD	20 (2.4%)	23.1 (6.69–79.7)	1.8 × 10^–12^	5.95 (1.36–26.2)	0.02
Asthma	38 (4.5%)	0.55 (0.21–1.43)	ns		
**Neurological disease**	88 (10.6%)	5.81 (3.66–9.21)	4.5 × 10^–16^	3.22 (1.70–6.09)	3.4 × 10^–4^
Stroke	27 (3.3%)	6.16 (2.80–13.54)	3.5 × 10^–7^		
Neurological disease and stroke	69 (8.3%)	5.49 (3.30–9.14)	8.7 × 10^–13^		
Neurological disease other than stroke	61 (7.2%)	4.71 (2.76–8.04)	9.2 × 10^–10^		
Kidney diseases	59 (7.1%)	5.00 (2.90–8.59)	3.1 × 10^–10^	2.48 (1.19–5.15)	0.02
Disability**	137 (19.7%)	3.56 (1.79–7.07)	1.3 × 10^–4^		
**ACE or AT1 inhibitors**	264 (50.5%)	2.23 (1.46–3.41)	1.8 × 10^–4^		
ACE inhibitors	133 (25.4%)	1.78 (1.14–2.78)	0.01		
AT_1_ antagonists	133 (25.4%)	1.53 (0.97–2.39)	ns		

*ns* not significant.

*Percentages were calculated based on those individuals that answered the question.

**Disability of any kind was documented only in the COVER study, not the ViP study.

***Multivariate analyses were performed using binary logistic regression with forward inclusion.

Medications used to treat hypertension were also associated with the risk of hospitalization. ACE inhibitors and AT1-receptor blockers were associated with a similar risk increase (Table 3), but none of them was an independent predictor of risk of hospitalization.

Notably, disability of any grade was associated with a 3.1-fold increase in risk of hospitalization (Table 3). Only 3.9% of non-disabled participants were hospitalized, compared to 11.1% of disabled participants. However, these analyses were based only on the 36 hospitalized individuals from the outpatients study, as in the ViP study grade of disability was not documented.

## Discussion

In this study, we analyzed the frequency and time course of SARS-CoV-2 infection in a large cohort of outpatients. We focused on identification of symptoms and comorbidities as risk factors associated with hospitalization. The main findings are the delayed onset of specific symptoms such as loss of smell and loss of taste. Most importantly, our data suggest that individuals reporting loss of taste but not loss of smell are more likely to suffer from progression of infection leading to hospitalization.

Loss of smell and loss of taste are regarded as relatively specific symptoms and therefore potentially useful in the differential diagnosis of SARS-CoV-2 infection^15^. Loss of smell or taste were among the five most commonly reported symptoms in the present study (Table 2). However, only 17.1% of our participants reported either of the two symptoms on the first day and 42.1% did not report loss of taste or loss of smell during the entire 25-day observation period (Fig. 3). This is in line with previously published outpatient studies^17,18^ and suggests that loss of smell or taste may be less useful as early markers of SARS-CoV-2 infection and PCR testing should also be triggered by less specific symptoms in order to detect infections earlier.

Our results support previous findings that loss of smell may be associated with lower risk for hospitalization^19–21^. Moreover, our study collected data starting at the early stages of the infection and may be less prone to underreporting of mild symptoms, which may occur at the time of hospitalization^22,23^.

Interestingly, our results indicate that isolated loss of taste without loss of smell predicts a higher risk of hospitalization (Fig. 4). To the best of our knowledge, this is the first report considering anosmia and ageusia as separate risk factors for hospitalization due to SARS-CoV-2 infection. An explanation may be found in the respective anatomy of the olfactory and gustatory sensory systems. The former consists of the first cranial nerve directly projecting to supratentorial structures, whereas the latter includes the nucleus of the solitary tract as first relay in the brainstem before moving upwards to the thalamus and the cortex^24^. Crucially, the nucleus of the solitary tract is part of the brainstem network regulating the respiratory and cardiac functions^25,26^. Therefore, loss of taste but not the loss of smell may be a direct marker of the SARS-CoV-2-induced brainstem damage contributing to the respiratory dysfunction in COVID-19^27,28^. We observed that loss of taste without loss of smell was significantly associated with dyspnea and nausea (Fig. 5), which further supports this hypothesis. Despite the biological plausibility of our results, independent replication is needed because of the limited number of hospitalized individuals in our study. Nevertheless, our data clearly point to separate consideration of both symptoms.

Our study emphasizes the multi-organ manifestations of SARS-CoV-2 infection. The increased risk for hospitalization we observed in participants with pulmonary symptoms like dyspnea (Fig. 4) or pulmonary comorbidities like COPD (Table 3) is well described^29–36^. However, gastrointestinal symptoms like vomiting and cardiovascular and renal comorbidities also increased the risk for hospitalization. This is in line with previously reported data both about inpatients^37–39^ and outpatients^6,40–43^.

Notably, not only neurological symptoms, but also neurological comorbidities were among the strongest risk factors for hospitalization in our study (Table 3). We observed a 6.2-fold increased risk for hospitalization in infected individuals with a history of stroke. Respiratory infections are known to be more common in patients after stroke^44–46^, possibly due to brain-induced immunodepression^47^. Alternatively, post-stroke patients are at higher risk for deep vein thrombosis and pulmonary embolism, which may be exacerbated by SARS-CoV-2 infection and lead to hospitalization or death^48,49^. However, neurological comorbidities other than stroke were almost equally strongly associated with the risk for hospitalization (Table 3).

Disability was among the strongest predictors of hospitalization after SARS-CoV-2 infection in our study (Table 3). This confirms previous findings^34^ and may reflect that multiple comorbidities including both chronic disease and health conditions like Down syndrome, or mental disability increase the risk of hospitalization^39,50,51^. Whatever the underlying cause(s), our finding suggests that disability in general rather than the presence of a single comorbidity may be better for identifying individuals who may benefit from targeted preventive measures against SARS-CoV-2.

The association between comorbidities and hospitalization, which we observed in this study (Table 3), are in line with previous studies reporting hypertension^36,52^, diabetes^42,53,54^, COPD^55,56^ or kidney disease^34,57^ as risk factors for severity or death with COVID-19. Recently developed scoring systems for outpatients identified increase age, comorbidities and symptoms like dyspnea and fever to be predictable for increased risk for hospitalization in outpatients^58,59^. This is in line with our data too.

Our study has several limitations. One limitation is a consequence from the very dynamic development of the pandemic. We recruited participants during the second wave of the infection, which in Germany was caused by the initial strain of the virus and therewith may be not directly transferable to the current infections with the delta or omicron strain. This limitation impacts not just our study but the majority of the currently published studies that collected data during the first wave of the pandemic^17,40–42,60^.

Because of the outpatient setting, we were unable to collect biological samples which would be of interest for some risk scores.

Another clear limitation is the questionnaire design of the study, which relies on self-reporting and thus may not perfectly reflect actual frequencies of different symptoms. We also did not ask about the intensity of the symptoms. However, without the possibility to apply precise and accurate standardized measurements, self-described severity of the symptoms is subjective and may be of limited use.

In conclusion, our study of SARS-CoV-2 patients with mild and moderate course of infection confirms the delayed onset of loss of smell and loss of taste and strengthens dyspnea and vomiting as symptoms associated with an increased risk of hospitalization. Our data also suggest that disability in general may be a useful marker for increased risk of hospitalization. Finally and most importantly, although preliminary, our data points to different roles of loss of smell and loss of taste as predictors for the severity of the infection.

## Supplementary Information

Supplementary Information.

## Data Availability

The data contains sensitive information about the health of the study participants that cannot be deposited openly. However, individual-level data that support the findings of this study could be obtained from corresponding author following an approval of the steering committee of the University Medicine Greifswald.
