# Supplementary material for: Course of disease and risk factors for hospitalization in outpatients with a SARS-CoV-2 infection
Source: Sci Rep. 2022 May 4;12:7249. doi: 10.1038/s41598-022-11103-0 (PMC9065670; doi:10.1038/s41598-022-11103-0)
Supplement: Supplementary file 1 — Supplementary Information. [file 41598_2022_11103_MOESM1_ESM.pdf]

Supplementary figures and tables

Course of disease and risk factors for hospitalization in outpatients with a SARS-CoV-2 infection

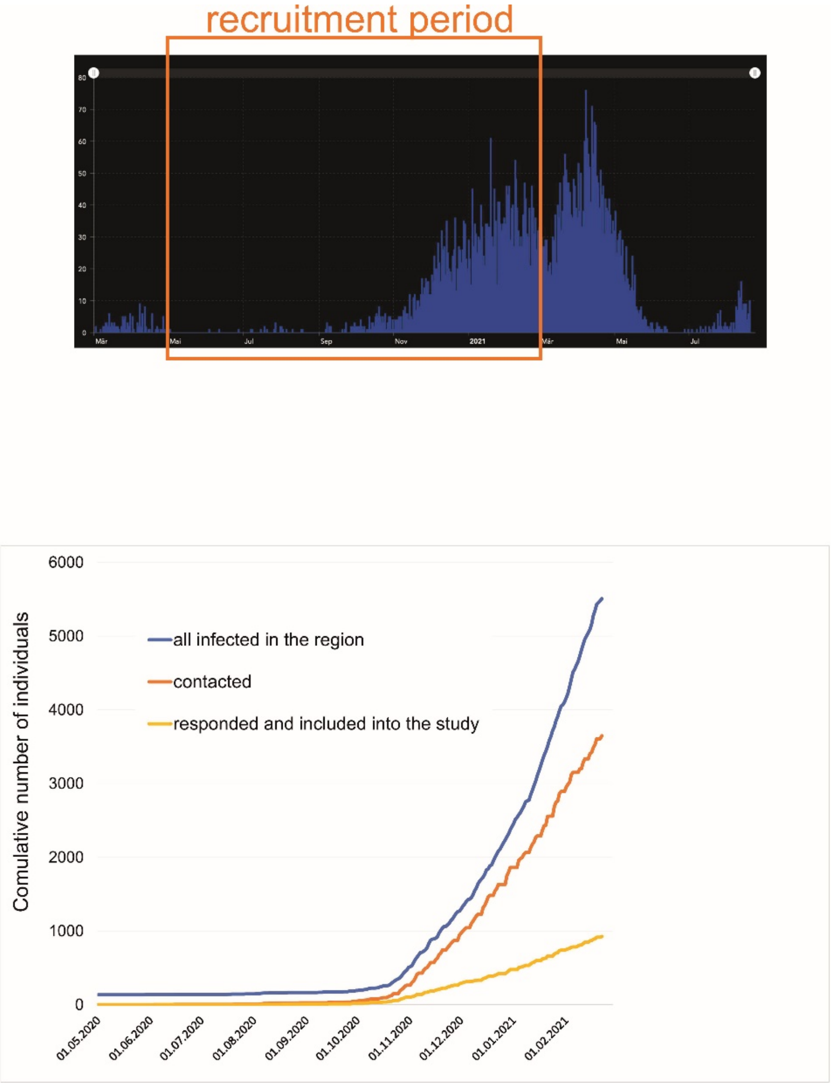

Supplementary figure 1 Time period and statistics of recruitment in the outpatients study

Upper panel shows the recruitment period relatively to the frequency of PCR-confirmed SARS-CoV-2 infections in the district of Vorpommern-Greifswald. Number of infections in the region is based on data from Robert Koch Institute (RKI) in Germany.

Lower panel shows cumulative numbers of individuals included into the study in relation to the cumulative number of individuals infected in the region and cumulative number of individuals contacted and asked to participate.

	General symptoms					Gastrointestinal symptoms				Pulmonary symptoms			Neurological symptoms		Other			
	Days with headache	Days with sore throat	Days with fatigue	Days with myalgia/Arthralgia	Days with fever > 37.9°C	Days with diarrhea	Days with vomiting	Days with abdominal Pain	Days with nausea	Days with dyspnea	Days with dry cough	Days with productive cough	Days with loss of taste	Days with loss of smell	Days with skin manifestation	Days with conjunctivitis		
	1	0.33	0.51	0.45	0.18	0.17	0.07	0.35	0.31	0.32	0.24	0.20	0.26	0.26	0.14	0.17	Days with headache	General symptoms
		1	0.33	0.26	-0.02	0.06	0.05	0.15	0.19	0.25	0.22	0.11	0.14	0.16	0.08	0.10	Days with sore throat	
			1	0.56	0.23	0.24	0.13	0.27	0.33	0.43	0.38	0.26	0.23	0.21	0.14	0.10	Days with fatigue	
				1	0.25	0.23	0.07	0.27	0.27	0.36	0.23	0.22	0.17	0.15	0.14	0.10	Days with myalgia/Arthralgia	
					1	0.13	0.08	0.14	0.17	0.13	0.13	0.12	0.05	-0.03	0.10	0.02	Days with fever > 37.9°C	
						1	0.27	0.35	0.19	0.21	0.14	0.24	0.07	0.04	0.07	0.01	Days with diarrhea	Gastrointestinal symptoms
							1	0.18	0.23	0.08	0.14	0.05	0.01	0.00	0.00	-0.02	Days with vomiting	
								1	0.44	0.20	0.14	0.20	0.08	0.09	0.18	0.10	Days with abdominal Pain	
									1	0.23	0.23	0.19	0.16	0.11	0.12	0.01	Days with nausea	
										1	0.30	0.25	0.12	0.12	0.13	0.07	Days with dyspnea	Pulmonary symptoms
											1	0.07	0.07	0.11	0.08	0.04	Days with dry cough	
												1	0.12	0.09	0.11	0.03	Days with productive cough	
													1	0.83	0.09	0.06	Days with loss of taste	Neurological symptoms
														1	0.05	0.08	Days with loss of smell	
															1	0.05	Days with skin manifestation	Other
																1	Days with conjunctivitis	

Supplementary figure 2. Correlations of the duration of the symptoms.

Pairwise correlations of the duration of all symptoms. The symptoms were stratified into groups depending on their type. The Pearson correlation coefficient is given and correlations reaching 2-sided significance level lower than $P=1 \times 10^{-13}$ are highlighted (for comparison: applying Bonferroni correction for 120 independent tests represented in this table gives a significance threshold of $p=4x10^{-4}$).

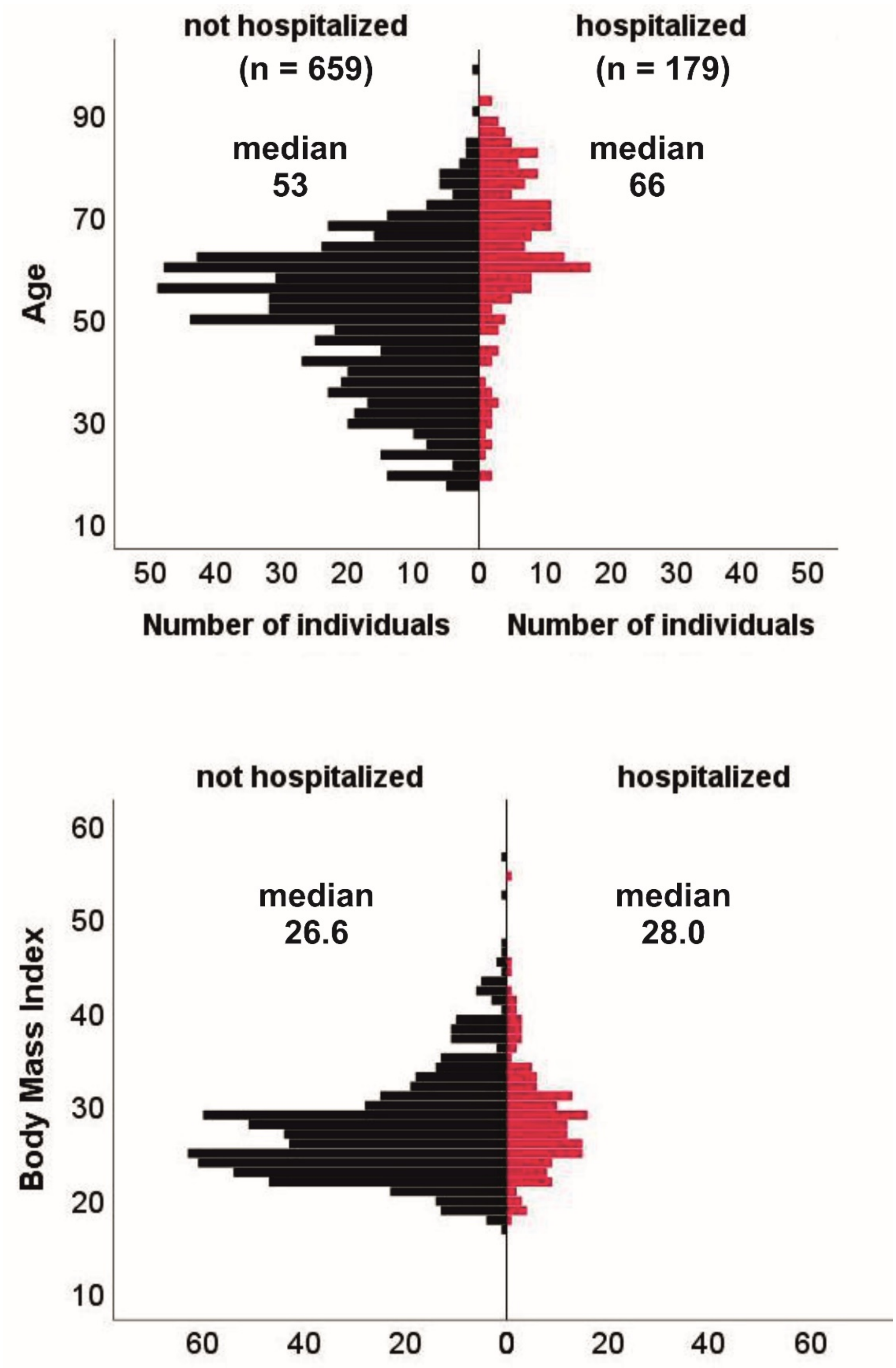

Supplementary figure 3. Differences in age (upper panel) and BMI (lower panel) distribution between the hospitalized and non-hospitalized study participants. Compared are the distributions of age between hospitalized (red) and non-hospitalized (black) study participants. Given are the absolute numbers and the corresponding median for both variables. Please, consider that the inclusion criteria for the both studies were 18 years of age or older

Supplementary table 1. Frequency of the initial symptoms

Initial Symptom	Number of individuals	%
Fatigue	428	60.28
Headache	345	48.59
Dry cough	290	40.85
Arthralgia/Myalgia	327	46.06
Loss of smell	109	15.35
Loss of taste	97	13.66
Sore throat	208	29.30
Productive cough	59	8.31
Diarrhea	80	11.27
Dyspnea	78	10.99
Nausea	80	11.27
Abdominal pain	62	8.73
Fever ≥ 38°C	88	12.39
Skin manifestation	18	2.54
Conjunctivitis	18	2.54
Vomiting	10	1.41

Supplementary table 2. Sex dependence of SARS-CoV-2 related symptoms

Symptom	Men (%)	Women (%)	p-value	OR (CI 95%)
Fatigue	79.2	86.6	0.01	1.69 (1.12 - 2.55)
Headache	64.8	80.0	2.40×10^{-05}	2.18 (1.53 - 3.10)
Dry cough	66.8	71.1	0.128	1.22 (0.87 - 1.71)
Myalgia/Arthralgia	64.9	69.8	0.141	1.25 (0.90 - 1.75)
Loss of smell	48.4	65.5	2.50×10^{-05}	2.02 (1.46 - 2.80)
Loss of taste	46.2	60.6	4.63×10^{-04}	1.79 (1.30 - 2.47)
Sore throat	42.0	57.6	1.42×10^{-04}	1.88 (1.36 - 2.59)
Productive cough	36.0	43.6	0.094	1.38 (0.99 - 1.91)
Diarrhea	33.3	43.3	0.02	1.53 (1.09 - 2.13)
Dyspnea	29.6	40.3	0.007	1.60 (1.14 - 2.25)
Nausea	23.6	39.9	2.70×10^{-05}	2.15 (1.50 - 3.08)
Abdominal pain	20.7	35.1	1.32×10^{-04}	2.08 (1.43 - 3.03)
Fever ≥ 38°C	34.2	35.4	0.480	1.06 (0.73 - 1.54)
Skin manifestation	9.2	13.9	0.084	1.60 (0.94 - 2.72)
Conjunctivitis	5.9	11.2	0.029	2.02 (1.07 - 3.80)
Vomiting	4.2	9.3	0.023	2.36 (1.14 - 4.88)

Supplementary table 3. Sex-dependency of symptom duration Shown are mean and standard deviations (SD) of the days with symptom. Included are only individuals who reported at least one day with this symptom.

**Symptom**	**Men**	**Women**	**p-value***
Fatigue	10.46 (6.49)	12.99 (7.29)	5.3×10^{-05}
Headache	6.67 (4.80)	7.96 (5.52)	0.015
Dry cough	9.02 (5.93)	11.52 (7.13)	4.27×10^{-04}
Myalgia/Arthralgia	7.58 (5.26)	7.82 (5.55)	0.699
Loss of smell	9.86 (6.85)	12.38 (6.71)	1.85×10^{-04}
Loss of taste	9.06 (6.15)	11.35 (6.61)	0.001
Sore throat	5.85 (4.50)	6.47 (5.53)	0.793
Productive cough	7.94 (6.18)	9.37 (6.84)	0.158
Diarrhea	4.12 (3.33)	3.69 (2.99)	0.201
Dyspnea	10.31 (7.52)	11.63 (7.63)	0.266
Nausea	4.03 (3.83)	4.78 (3.79)	0.055
Abdominal pain	4.86 (5.08)	4.56 (4.57)	0.870
Fever ≥ 38°C	3.60 (3.08)	2.98 (2.31)	0.179
Skin manifestation	7.77 (7.25)	8.06 (5.74)	0.443
Conjunctivitis	3.93 (2.30)	5.80 (2.22)	0.330
Vomiting	1.50 (0.71)	2.36 (4.86)	0.397

* two-tailed significance using nonparametric Mann-Whitney-U test

Supplementary table 4 Age dependence of symptom duration. Shown are mean and standard deviations (SD) of the day with the symptom. All participants with the respective symptom were analyzed.

**Symptom**	**Age 18 - 39**	**Age 40 - 59**	**Age >60**	**p-value**
Fatigue	10.23 (6.67)	12.35 (7.34)	12.95 (6.84)	0.001
Headache	7.04 (5.06)	7.57 (5.60)	7.84 (5.05)	0.343
Dry cough	8.49 (5.80)	11.19 (6.89)	11.19 (7.14)	0.002
Myalgia/Arthralgia	5.93 (4.36)	7.68 (5.46)	9.22 (5.79)	6.4×10^{-7}
Loss of smell	11.52 (6.43)	11.61 (6.93)	11.49 (7.32)	0.927
Loss of taste	10.92 (6.39)	10.29 (6.65)	10.69 (6.59)	0.558
Sore throat	6.08 (5.24)	6.44 (5.67)	6.15 (4.32)	0.656
Productive cough	6.46 (4.88)	9.28 (6.53)	10.35 (7.60)	0.004
Diarrhea	3.24 (2.70)	3.71 (3.05)	4.40 (3.43)	0.061
Dyspnea	10.05 (7.35)	12.38 (7.90)	10.66 (7.31)	0.168
Nausea	3.65 (2.93)	4.42 (4.13)	5.66 (3.80)	0.004
Abdominal pain	4.21 (4.27)	4.04 (4.08)	5.69 (5.40)	0.044
Fever ≥ 38°C	2.21 (1.67)	2.96 (2.07)	3.89 (3.29)	0.006
Skin manifestation	6.79 (4.80)	6.59 (5.42)	13.36 (7.41)	0.004
Conjunctivitis	4.00 (2.83)	6.27 (5.09)	3.64 (2.16)	0.211
Vomiting	1.38 (0.52)	2.32 (2.54)	2.47 (2.62)	0.511

* two sided significance using nonparametric Kruskal-Wallis test

Supplementary table 5 Age dependence of the risk to experience SARS-CoV-2 related symptoms

**Symptom**	**Age 18 - 39**	**Age 40 - 59**	**Age >60**	**p-value**
Fatigue	82.2	85.4	82.5	0.570
Headache	80.7	78.2	63.2	$7{,}9 \times 10^{-5}$
Dry cough	68.1	66.3	74.4	0.148
Myalgia/Arthralgia	68.1	68.2	76.3	0.978
Loss of smell	68.9	62.7	44.2	2×10^{-6}
Loss of taste	60.2	60.3	42.4	1.8×10^{-4}
Sore throat	63.4	52.2	40.4	8×10^{-5}
Productive cough	40.6	41.9	38.3	0.739
Diarrhea	39.9	36.7	42.4	0.459
Dyspnea	37.3	34.6	37.0	0.802
Nausea	32.5	34.4	32.2	0.862
Abdominal pain	26.9	31.1	29.1	0.648
Fever ≥ 38°C	28.4	32.9	41.2	0.059
Skin manifestation	15.8	12.8	7.8	0.076
Conjunctivitis	6.6	12.5	6.2	0.036
Vomiting	5.2	7.2	8.6	0.499

*p-value Pearson's chi-squared test

Supplementary table 6. Basic characteristics and risk factors at baseline of the ViP study population Shown are median (first and third quartile) for age, BMI and absolute number (prevalence) for the remaining variable. Percentage calculated by known number of each risk factor.

**Parameter**	**All (n=143)**	**Male (n=88)**	**Female (n=55)**	**p-value***
Age in years	66 (25 - 92)	66.5 (25 – 92)	66 (25 – 92)	0.535
BMI in kg/m^2	27.9 (17.6 – 54.2)	27.17 (17.6– 42.5)	28.06 (18.9 – 54.2)	0.585
Comorbidity				
• Hypertension	83 (59.7%)	54 (63.5%)	29 (53.7%)	0.250
• Joint diseases	14 (10.4%)	9 (10.8%)	5 (9.6%)	0.820
• Cardiovascular diseases	52 (38.0%)	28 (33.3%)	24 (45.3%)	0.160
• Pulmonary diseases	35 (25.2%)	22 (25.6%)	13 (24.5%)	0.890
• Diabetes mellitus	35 (25.2%)	21 (24.7%)	14 (25.9%)	0.872
• Neoplasia	17 (12.2%)	12 (14.3%)	5 (9.1%)	0.361
• Neurological diseases	39 (29.3%)	25 (30.9%)	14 (26.9%)	0.626
• Liver diseases	14 (10.4%)	11 (13.4%)	3 (5.7%)	0.149
• Kidney diseases	26 (19.3%)	16 (19.0%)	10 (19.6%)	0.936
• Peripheral artery disease	3 (2.2%)	3 (3.6%)	0 (0%)	0.283

* for differences between male and female participants (calculated using Pearson χ^2 test except for age, BMI where Mann-Whitney U test was used, using Fisher´s exact test for peripheral artery disease)
